# Optimizing Patient Education in Emergency Medicine Overutilization

**DOI:** 10.51894/001c.144152

**Published:** 2025-09-30

**Authors:** Destiny Kanning

**Affiliations:** 1 College of Osteopathic Medicine Michigan State University; 2 Emergency Medicine UM Health Sparrow

## 48

The overutilization of pediatric emergency departments (EDs) for non-emergent health concerns is a persistent issue, particularly among economically disadvantaged populations. This phenomenon often results from limited health literacy and inadequate access to primary care resources. The study aims to investigate the root causes of this overutilization and evaluate the effectiveness of patient education strategies in mitigating the burden on emergency services in Michigan, and specific local communities around the university.

Data were collected from Sparrow Health Systems, Motts Children’s Hospital, and UofM Health West emergency departments over two years. A mixed-methods approach was employed, combining quantitative analysis of ED visit records with qualitative surveys conducted among caregivers. The study also implemented an educational intervention targeting common misconceptions about when to seek emergency care. Outcomes were assessed based on changes in caregiver-reported health literacy and ED visit frequencies over 2 month periods.

Preliminary results indicate that targeted educational interventions have the potential to reduce non-emergent ED visits among participants by 68%. Over 2000+ patients and their families reported improved confidence in recognizing urgent versus non-urgent conditions and an increased likelihood of utilizing primary care resources in the local community.

These findings highlight the potential of tailored patient education to alleviate the overuse of pediatric EDs, especially in underserved communities. By addressing barriers related to language access and health literacy, the study demonstrates a pathway to improving healthcare equity and resource allocation in Michigan.

Optimizing patient education is a scalable and effective strategy for reducing non-emergent ED visits. This approach aligns with broader efforts to build a healthier and more equitable healthcare system by empowering families with the knowledge and tools needed to make informed decisions.

